# Multivessel Coronary Artery Disease in a Patient Treated With Tofacitinib: A Case Report

**DOI:** 10.7759/cureus.86235

**Published:** 2025-06-17

**Authors:** Wongelawit Zerihun, Joud Fahed, Danhue Moodie, Fana Cisse-Diouf

**Affiliations:** 1 Department of Internal Medicine, Ascension Saint Agnes Hospital, Baltimore, USA; 2 Department of Internal Medicine, Ross University School of Medicine, Miramar, USA

**Keywords:** cardiovascular risk (cvr), coronary angiography, coronary artery disease, jak inhibitors, tofacitinib

## Abstract

Tofacitinib is a second-generation selective Janus kinase (JAK) inhibitor targeting the JAK1 enzyme, which is used to treat several autoimmune diseases, including rheumatoid arthritis (RA), psoriatic arthritis, ankylosing spondylitis, idiopathic arthritis, and ulcerative colitis. JAKs are enzymes that play a key role in inflammatory and immune signaling pathways. The medication is particularly useful as it can be given orally. However, over time, there has been much data that links this drug with an increased risk of cardiovascular events. We present a case of a 73-year-old male with a history of RA and hypertension presenting to the hospital with typical cardiac chest pain. He was found to have ischemic EKG changes and elevated troponin levels. He then underwent cardiac catheterization, which revealed significant coronary artery disease.

## Introduction

Tofacitinib represents a significant advancement in the treatment of autoimmune conditions as an oral targeted synthetic disease-modifying antirheumatic drug (tsDMARD). tsDMARDs are synthetic medications (non-biologic) that specifically target certain inflammatory pathways in the immune system, like Janus kinase (JAK) inhibitors. Rheumatoid arthritis (RA) is characterized by chronic inflammation driven by pro-inflammatory cytokines such as interleukin-6 (IL-6) and interferon-gamma (IFN-γ), which activate intracellular JAKs through immune cell receptors. This triggers the JAK-STAT (signal transducers and activators of transcription) signaling pathway, leading to inflammatory gene expression and immune cell activation. JAK inhibitors, such as tofacitinib, baricitinib, and upadacitinib, block specific JAK enzymes (JAK1, JAK2, JAK3, TYK2), thereby preventing STAT activation and reducing the production of inflammatory mediators. This disruption decreases immune cell recruitment to joints and mitigates inflammation in RA. Following its initial FDA approval in 2012 for adults with refractory RA, the drug’s therapeutic applications expanded to include psoriatic arthritis (2017) and ulcerative colitis (2018) [1]. The oral administration route of tofacitinib offered a distinct advantage over injectable biologics, contributing to its widespread adoption and leading to its endorsement by the European League Against Rheumatism (EULAR) in 2019 as a first-line treatment option for patients who failed conventional DMARD therapy, such as methotrexate [2,3]. Despite its therapeutic benefits, increasing evidence has raised significant concerns about tofacitinib’s cardiovascular safety profile. Clinical trials and post-marketing surveillance data have consistently demonstrated an increased risk of cardiovascular events among users, particularly in those with pre-existing cardiovascular disease or risk factors.

We present a case highlighting these cardiovascular concerns: a patient with RA who developed acute coronary syndrome after two years of tofacitinib therapy. Despite having only well-controlled hypertension as a cardiac risk factor, the patient presented with chest pain and was diagnosed with ST-elevation myocardial infarction (STEMI) and managed with revascularization therapy. This case underscores the importance of careful cardiovascular risk assessment and monitoring in patients receiving tofacitinib therapy, even those without a significant cardiac history.

## Case presentation

The patient is a 73-year-old Caucasian male with a significant past medical history of RA and well-controlled hypertension who presented with a sudden onset of left-sided retrosternal chest tightness discomfort associated with intermittent radiation to his left arm and exertional dyspnea of five hours duration. He endorsed associated diaphoresis and nausea; he, however, denied any palpitations, lightheadedness, or a personal or family history of similar illness, reported no diabetes, smoking, or alcohol use.

The patient’s past medical history is notable for longstanding RA; he had previously been treated with methotrexate; however, due to inadequate disease control (Disease Activity Score-28 (DAS28) in RA, reportedly as high as 6), he was switched to tofacitinib monotherapy two years prior to this presentation. The patient reported current good RA disease control and had not been requiring nonsteroidal anti-inflammatory drugs (NSAIDs) for symptom relief, and his history was also remarkable for hypertension, which was well managed with losartan 75 mg once daily.

**Figure 1:**
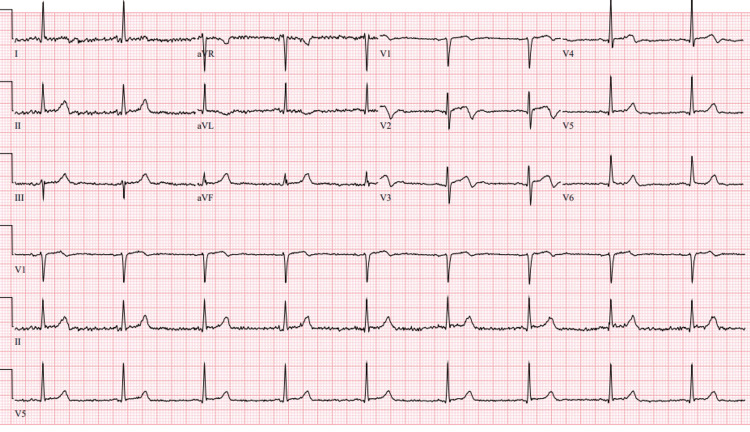
A 12-lead ECG showing sinus bradycardia with subtle ST elevations in the anterior leads (V1-V3) and biphasic T-waves in the anterolateral leads.

On examination, he appeared to be in acute distress due to the pain. His heart rate was 57 beats per minute (bpm), and he had a blood pressure of 164/89 mm Hg. His oxygen saturation was 98% on room air. Further examination revealed clear lungs on auscultation, with no jugular venous distension, murmurs, and peripheral edema. High-sensitivity troponin levels were 1664 ng/L and 3600 ng/L at zero and one hour, respectively. The lipid panel showed a total cholesterol of 119 mg/dL, triglycerides of 52 mg/dL, low-density lipoprotein (LDL) of 61 mg/dL, and high-density lipoprotein (HDL) of 48 mg/dL (Table 1). A chest X-ray was also obtained, which showed a normal-sized heart with no consolidation or abnormality noted in the lungs. A serial 12-lead ECG showed sinus bradycardia with dynamic ST changes in the anterior and septal leads (Figure 1), which led to immediate cardiac catheterization laboratory activation.

**Table 1:** Laboratory test results with reference ranges LDL: low-density lipoprotein; HDL: high-density lipoprotein

Test	Result	Reference range
High-sensitivity troponin (baseline)	1664 ng/L	0-35 ng/L
High-sensitivity troponin (one hour)	3600 ng/L	0-35 ng/L
Total cholesterol	119 mg/dL	<200 mg/dL
Triglycerides	52 mg/dL	<150 mg/dL
LDL	61 mg/dL	<100 mg/dL
HDL	48 mg/dL	>50 mg/dL
Creatinine	0.8 mg/dL	0.72-1.25 mg/L

**Figure 2:**
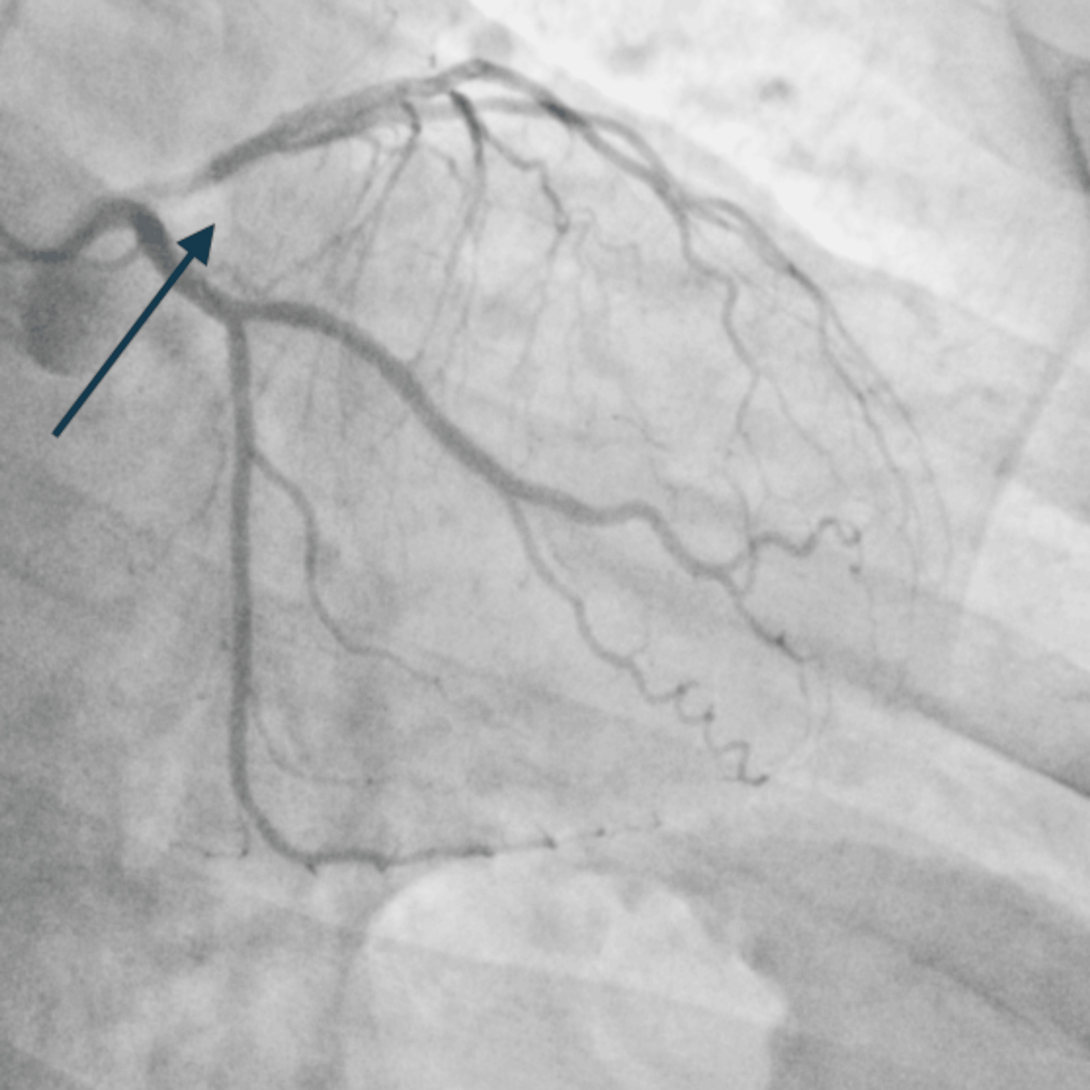
Left anterior oblique (LAO) view showing 99% occlusion of the mid left anterior descending artery (LAD), with notable ramus and circumflex marginal occlusions (arrow).

**Figure 3:**
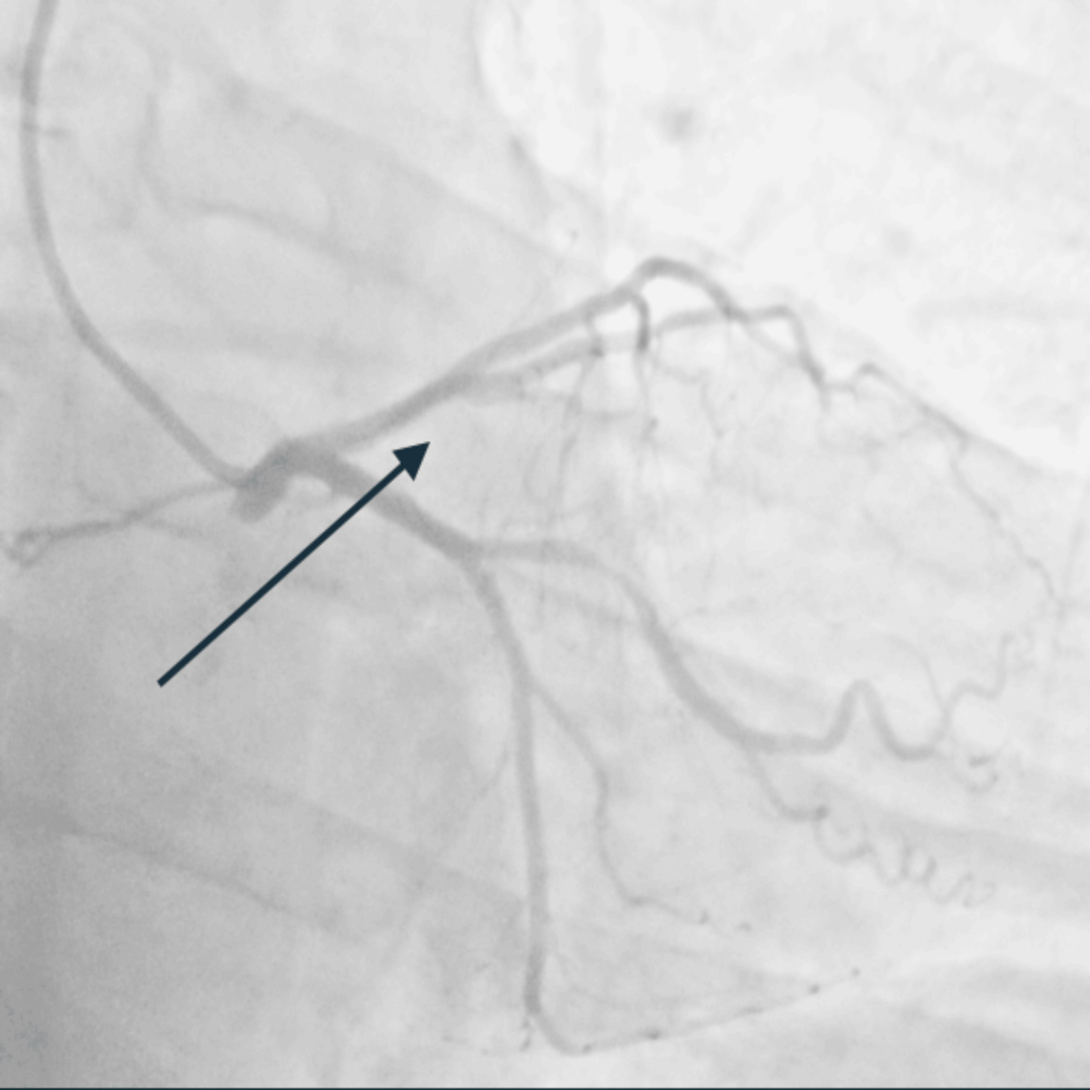
Successful placement of drug-eluting stent in the LAD and ramus with excellent flow and reperfusion (arrow). LAD: left anterior descending artery

Coronary angiography revealed right-dominant circulation with a normal left main coronary artery, 99% occlusion of the mid left anterior descending artery (LAD), 90% ramus intermedius, 80% circumflex marginal branch, and 80% right coronary artery (Figure 2). A drug-eluting stent was successfully placed in the LAD and ramus with excellent flow and reperfusion (Figure 3). On ventriculogram, ejection fraction (EF) was noted to be 55% with a left ventricular end diastolic pressure (LVEDP) of 3 mmHg. The patient underwent successful percutaneous coronary intervention (PCI) of the LAD and the ramus. He was initially loaded with cangrelor and aspirin and maintained on ticagrelor and aspirin post-PCI. A staged PCI approach was employed per institutional protocol. He subsequently returned to the catheterization lab for intervention of the proximal circumflex marginal and mid-right coronary artery. LVEDP on repeat left heart catheterization was 18 (the initial low value was likely artifactual) and also showed widely patent stents in both the mid-LAD and ramus with no residual disease and Thrombolysis in Myocardial Infarction (TIMI) grade 3 flow.

Following completion of revascularization, a multidisciplinary discussion involving the primary medical team, cardiology, and rheumatology was held. Given the newly identified high cardiovascular risk and the accumulating evidence linking tofacitinib use with increased cardiovascular events, a decision was made to discontinue tofacitinib. The patient was advised to follow up with his primary rheumatologist for consideration of alternative disease-modifying therapy. He was discharged in stable condition on high-intensity statin therapy, aspirin, and ticagrelor, with a one-year planned dual antiplatelet therapy regimen. A follow-up appointment with cardiology was arranged.

## Discussion

Tofacitinib and the rest of the JAK inhibitors, through several clinical trials, have demonstrated efficacy comparable to TNF inhibitors in the treatment of RA. With direct effects on JAK pathways, as well as indirect actions with reductions in synovial phosphorylation of STAT1 and STAT3 proteins, it causes a significant reduction in symptom burden in these patients [3]. However, mounting concerns regarding its cardiovascular safety profile have emerged through various clinical trials and observational studies [4]. The mechanisms underlying these cardiovascular effects are still unclear and require further studies.

Tofacitinib targets JAK1 and JAK3, impairing cytokine signaling, and may contribute to a pro-atherogenic state. Although several potential mechanisms have been proposed, one widely accepted pathway is through alteration in lipid metabolism and inflammatory pathways [5]. Studies have shown that tofacitinib treatment leads to elevations in both LDL and HDL cholesterol levels, potentially disrupting the balance between pro- and anti-inflammatory factors and leading to endothelial dysfunction. Additionally, its influence on the renin-angiotensin-aldosterone system (RAAS), marked by increased angiotensin-converting enzyme (ACE) levels, may contribute to cardiovascular stress. Modulation of ACE2 expression has been observed, although its role remains more associative than mechanistic or predictive. Current evidence does not support ACE/ACE2 alterations as reliable clinical biomarkers for cardiovascular risk in JAK inhibitor users [6].

A prospective cohort study showed that one-year tofacitinib therapy increased ACE levels after six and 12 months, while ACE2 activity only transiently increased at six months. The ACE/ACE2 ratio increased after one year of therapy (p < 0.05) [7]. The ORAL Post-market Surveillance study (2014-2020), involving 4,362 patients aged 50 and older with at least one cardiovascular risk factor, administered either 5 mg or 10 mg of tofacitinib twice daily or a TNF inhibitor (adalimumab or etanercept), having followed the subjects over a median of 4.0 years, revealed a higher incidence of major adverse cardiovascular events (MACEs) in tofacitinib groups (3.4%) compared to TNF inhibitors (2.5%) with a hazard ratio of 1.33 [8,9].

These findings were further corroborated by a 2023 post hoc analysis, which demonstrated higher MACE incidence rates in tofacitinib-treated patients with psoriatic arthritis and psoriasis who have a baseline elevated atherosclerotic cardiovascular disease (ASCVD) risk. A relatively low number of MACE was observed overall; however, patients who at baseline had a low 10-year risk of ASCVD had a lower MACE incidence rate (95% confidence interval (CI) (0.08 (0.00, 0.42)) than patients with higher cardiovascular risk categories. The highest MACE incidence rate (1.37) was in patients with a high (≥20%) 10-year risk of ASCVD. While the STAR-RA study (2022) showed more modest risk increases in real-world settings (hazard ratio: 1.01, 95% CI: 0.83-1.23), it still suggested potentially higher risks in patients with pre-existing cardiovascular conditions [9].

Although tofacitinib has shown the strongest association with cardiovascular events, other JAK inhibitors, such as baricitinib (JAK1/2) and upadacitinib (selective JAK1), have also been linked to modest lipid increases. However, MACE rates in trials such as RA-BEAM and RA-BUILD have not demonstrated the same magnitude of risk as tofacitinib [10].

## Conclusions

This case contributes to the growing body of evidence underscoring the need for a more nuanced and individualized approach when initiating tofacitinib therapy. While large clinical trials have highlighted an increased incidence of MACE among high-risk individuals receiving JAK inhibitors, this case draws attention to the possibility that significant cardiovascular complications may also occur in patients without traditional risk factors. This reinforces the importance of maintaining a high index of suspicion and the need for cardiovascular risk assessment not only at baseline but at regular intervals throughout the course of JAK inhibitor therapy. Incorporating routine screening tools, such as electrocardiograms, lipid panels, and stress testing, into the clinical monitoring of patients on agents like tofacitinib may help identify subclinical cardiovascular disease and mitigate potential adverse outcomes.

Lastly, this case underscores the critical role of interdisciplinary collaboration among rheumatology, cardiology, and primary care in ensuring comprehensive, safe, and effective management of patients on immunomodulatory therapies. As evidence continues to evolve, clinicians must remain attentive to emerging data and adapt therapeutic strategies accordingly, balancing the benefits of disease control with vigilance for potential systemic risks.
